# Comparison of the Functional State and Motor Skills of Patients after Cerebral Hemisphere, Ventricular System, and Cerebellopontine Angle Tumor Surgery

**DOI:** 10.3390/ijerph19042308

**Published:** 2022-02-17

**Authors:** Stanisław Krajewski, Jacek Furtak, Monika Zawadka-Kunikowska, Michał Kachelski, Marcin Birski, Marek Harat

**Affiliations:** 1 Department of Physiotherapy, University of Bydgoszcz, Unii Lubelskiej 4, 85-059 Bydgoszcz, Poland; 2 Department of Neurosurgery, 10th Military Research Hospital and Polyclinic, 85-681 Bydgoszcz, Poland; jacek.furtak2019@gmail.com (J.F.); kachelskim@gmail.com (M.K.); mbirski@poczta.fm (M.B.); harat@10wsk.mil.pl (M.H.); 3 Franciszek Łukaszczyk Oncology Center, Department of Neurooncology and Radiosurgery, 85-796 Bydgoszcz, Poland; 4 Department of Human Physiology, LudwikRydygier Collegium Medicum in Bydgoszcz Nicolaus Copernicus University in Torun, Karłowicza 24, 85-092 Bydgoszcz, Poland; m.zkunikowska@cm.umk.pl; 5 Department of Neurosurgery and Neurology, LudwikRydygier Collegium Medicum in Bydgoszcz Nicolaus Copernicus University in Torun, M. Sklodowskiej-Curie 9, 85-094 Bydgoszcz, Poland

**Keywords:** brain tumor, cerebral hemisphere, cerebellopontine angle, function, rehabilitation, ventricular system

## Abstract

Brain tumor location is an important factor determining the functional state after brain tumor surgery. We assessed the functional state and course of rehabilitation of patients undergoing surgery for brain tumors and assessed the location-dependent risk of loss of basic motor skills and the time needed for improvement after surgery. There were 835 patients who underwent operations, and 139 (16.6%) required rehabilitation during the inpatient stay. Karnofsky Performance Scale, Barthel Index, and the modified Rankin scale were used to assess functional status, whereas Gait Index was used to assess gait efficiency. Motor skills, overall length of stay (LOS) in hospital, and LOS after surgery were recorded. Patients were classified into four groups: cerebral hemisphere (CH), ventricular system (VS), and cerebellopontine angle (CPA) tumors; and a control group not requiring rehabilitation. VS tumor patients had the lowest scores in all domains compared with the other groups before surgery (*p* < 0.001). Their performance further deteriorated after surgery and by the day of discharge. They most often required long-lasting postoperative rehabilitation and had the longest LOS (35 days). Operation was most often required for CH tumors (77.7%), and all metrics and LOS parameters were better in these patients (*p* < 0.001). Patients with CPA tumors had the best outcomes (*p* < 0.001). Most patients (83.4%) with brain tumors did not require specialized rehabilitation, and LOS after surgery in the control group was on average 5.1 days after surgery. VS tumor patients represent a rehabilitation challenge. Postoperative rehabilitation planning must take the tumor site and preoperative condition into account.

## 1. Introduction

Rehabilitation after brain tumor surgery is complex due to the diverse symptoms and neurological sequelae seen in these patients. Outcomes for brain tumor patients are influenced by many factors, including disease growth rate and duration, tumor size, grade of malignancy, and patient age. Tumor location has a particularly important impact on outcomes [1,2,3,4,5,6,7]. Approximately 60% of brain tumors—especially gliomas—are sited in the cerebral hemispheres, over half of which are highly malignant glioblastomas [1,4,6,7,8]. These can cause a constellation of symptoms depending on the affected lobe. Patients with frontal lobe tumors (~26%) may have limb paresis, apraxia, ataxia, and gait disorders requiring rehabilitation that may be disrupted by psycho-intellectual, emotional, social and cognitive, and speech disturbances. Parietal lobe lesions (12%) cause sensory disturbances, and those affecting the limbs and trunk or resulting in structural apraxia and body pattern disorders can significantly reduce performance. Cerebellar and cerebellopontine angle (CPA) tumors can cause balance and coordination disturbances and ataxia, which result in gait disorders, adiadochokinesis, and fatigue [1,8,9], and many patients with CPA tumors need rehabilitation for facial nerve palsy [10,11,12,13,14]. Tumors of the ventricular system (VS) are rare, accounting for 2–7% of all brain tumors [4,15,16], and can cause increased intracranial pressure and hydrocephalus. These patients require rehabilitation to improve balance and gait efficiency, but their course of rehabilitation is influenced by more frequent (20–36%) postoperative complications (bleeding into the ventricular system, edema, cerebrospinal fluid (CSF) leak, ventriculitis/meningitis) than tumors located at other sites, with new neurological deficits common after surgery at this location [15,16,17].

As survival after brain tumor surgery increases [18,19,20,21], so too does the role for rehabilitation in these patients [6]. There is strong evidence to support multidisciplinary rehabilitation for other neurological and oncological conditions but relatively little data on rehabilitation following brain tumor treatment [22,23]. Current assessments are not fully comprehensive and fail to take tumor site, functional state before surgery, the incidence of complications, and the impact of these factors on outcomes and length of hospital stay (LOS) after brain tumor surgery into account. LOS is therefore important to examine in detail, as it is a valid measure of quality of care and a universal metric gauging the success of hospital cost containment, cost reduction, and alternative care delivery systems [24]. While existing studies have compared the effects of surgery on function, the state before surgery, and outcomes at discharge, there are little data on patient condition immediately after surgery as a starting point for rehabilitation. Determining the risks associated with tumor site and preoperative condition might therefore be helpful for anticipating the need for and type of postoperative rehabilitation in neurosurgical wards and to identify and preemptively manage at-risk individuals.

We therefore specifically addressed these knowledge gaps in the current study. Our primary aim was to assess the functional status, motor skills, and gait efficiency of patients undergoing brain tumor surgery. An additional aim was to assess the incidence of complications affecting the rehabilitation course and time parameters such as the overall LOS, LOS after surgery, LOS in the Intensive Care Unit (ICU), the time needed for rehabilitation, and the time needed to improve any loss in basic motor skills after surgery.

## 2. Materials and Methods

### 2.1. Patient Cohort

The Bioethics Committee at the Military Medical Chamber approved the study protocol (no. 164/18). This was a single-center prospective, observational case-control study (three intervention groups and a control group) with follow-up time from the day of admission to the clinic to the day of discharge. There were 835 patients undergoing operations for brain tumors from August 2018 to February 2020 (18 months), 139 (16.6%) of whom required rehabilitation during their inpatient stay at the Neurosurgery Clinic. Three patients refused to participate in the study, and three died. The study therefore included 133 patients who underwent rehabilitation after brain tumor surgery. One-hundred and twenty-seven patients (95.5%) had tumors in the three most common locations: cerebral hemispheres (CH; *n* = 96), ventricular system (VS; *n* = 16), and cerebellopontine angle (CPA; *n* = 15). Surgery for all CPA tumors was via retrosigmoid access. Access to the remaining locations was selected individually to minimize damage to brain tissues. Head MRI with tractography was used to select access, and functional MRI was used in selected cases.The inclusion criteria were as follows: patients after brain tumor surgery; neurological deficits found; functional state worsened by surgery; and need for prolonged rehabilitation. 

A control group of 78 patients was also assessed in whom the physical therapist assisted in verticalization after surgery, secured the gait in the first few days after surgery, and educated patients on function in the early postoperative period. This group represented over 80% of all operated patients and was chosen to provide a reference for the results of the three study groups. While we were particularly interested in comparing the CH, VS, and CPA groups, comparing the results of these groups to the control group allowed us to assess the degree to which individual characteristics differed from patients whose baseline and postoperative status did not require rehabilitation. The inclusion criteria for the control group were as follows: patients after brain tumor surgery; no neurological deficits; no postoperative complications limiting activity and verticalization; functional state did not worsen after surgery; rehabilitation was not needed; and not banned from leaving bed.

### 2.2. Patient Assessment

Primary variables included functional status (Karnofsky Performance Scale—KPS, Barthel Index—BI, modified Rankin scale—MRS), motor skills (passive sitting, active sitting, standing, independent gait), and gait efficiency (Gait Index—GI). Activities of daily living (ADL) were assessed with the Barthel Index (BI), which assesses self-reliance in eating, self-transferring (e.g., from bed to wheelchair), maintaining personal hygiene, using the toilet, washing, moving on flat surfaces and stairs, dressing, and controlling urine and bowel motions. Each activity is scored 5, 10, or 15 up to a total of 100 points: 0–20 means a severe condition, 20–80 indicates that the patient requires help of various degrees, and >80 denotes an independent patient. The validity, reliability, and responsiveness of this scale have previously been reported, with its Pearson product moment correlation coefficient (PCC) ≥ 0.80 and Cronbach’s α coefficient or intraclass correlation (ICC) ≥ 0.70 denoting high test–retest and inter-rater reliability, respectively [25]. The BI scale was found to be structurally valid (r ≥ 0.50), and the scale was shown to detect clinically significant changes over time (i.e., responsive) [25].

General condition and performance living with cancer was assessed with the Karnofsky Performance Status Scale (KPS; scale with a 10-point gradation, where 100 points is full performance and 0 death). This scale assesses the impact of cancer on patients’ activity, taking into account their care and medical needs. A KPS score > 70 means that the patient can continue with normal activities and work with no special care needed; a KPS of 40–70 denotes that the patient is unable to work, can live at home, can care for most personal needs, but that a varying degree of assistance is needed; a KPS < 40 denotes that the patient is unable to care for self and requires the equivalent of institutional or hospital care, and the disease may be progressing rapidly [26]. In a study of cancer patients, the KPS PCC was 0.89 and the scale was considered to be highly reliable [27,28]. Inter-observer reliability for the KPS was 0.97 (Cronbach’s α coefficient). The KPS scale was also valid (r ≥ 0.70) [25,29].

The degree of dependence was assessed with amodified Rankin scale (MRS; a scale with 1-point increments, where 0 is no symptoms, and 6 is death). This scale assesses to what extent and in what time dimension the patient requires care. The MRS has been shown to have strong test–retest and inter-rater reliability in different studies (ĸ-weighted = 0.94, 0.99). With respect to validity, moderate to strong correlations (Pearson r = 0.60 to 0.86) have also been reported [30,31].

Gait efficiency was assessed with the 10-point scale (Gait Index—GI), graded in 1-point increments:

1. Impossible to achieve an upright vertical position;
2. Possible to stand with the assistance of the therapist who secures the knees, hips, and trunk;
3. Independent standing, the possibility of supporting with equipment;
4. Gait while learning to walk with a therapist, no the possibility of practical use;
5. Gait with the assistance of another person, but only within a room, accessing the wheelchair, toilet;
6. Gait with the assistance of another person, distance of several dozen meters (walking in the hospital corridor);
7. Independent gait with a walking frame;
8. Independent gait with crutches or walking stick;
9. Incorrect independent gait;
10. Correct independent gait.

The Bioethics Committee at the Military Medical Chamber approved this scale. This is a simple and easy-to-use scale used by us in the Neurosurgery Clinic to assess gait disorders in everyday practice. This scale has not yet been validated in patients with brain tumors.

Secondary variables included the overall LOS, the LOS after surgery, the LOS in the ICU, the number of rehabilitation days, and the incidence of complications affecting the rehabilitation course. The Landriel Ibañez four-grade classification was used to assess the severity and type of complications affecting the course of rehabilitation, where Grade I refers to non-life-threatening complications treated without invasive procedures; Grade II refers to complications requiring invasive management; Grade III refers to complications requiring treatment in the ICU; and Grade IV refers to death due to complications.

Assessments were performed by completing an assessment sheet. Each participant was given an individual sheet with an assigned number. In the first part of the assessment, data on hospital stay and surgery, medical record number, date of admission and discharge, date of surgery, number of days in the ICU, number of days of rehabilitation, tumor location, gender, and age were entered. This part was completed on the day of inclusion and was completed on upon discharge (endpoint). The discharge time was 2–90 days in the CH, VS, and CPA groups and 3–8 days after surgery in the control group. The second part of the assessment was about individual motor skills: passive sitting (the patient can spend time in a wheelchair), active sitting (independent sitting, stable trunk), independent standing, and independent gait with or without orthopedic equipment. The results were recorded before the operation on the day of inclusion and the day after surgery on which the patient was able to perform each of them. The third part of the assessment was completed three times: on the day of inclusion, immediately after surgery (day 2–3), and upon discharge. All ten skills assessed by the BI scale and the overall BI, KPS, MRS, and GI scores were noted based on observing and testing the patient according to each instrument. The final part of the assessment was concerned with the complications of the course of rehabilitation: the ability to independently walk with or without orthopedic equipment; which day after surgery the complication appeared; and for how many days it resulted in limiting exercise, verticalization, and leaving the bed. These data in part four were entered on a regular basis. 

The tests were performed directly by staff working with the patients in the neurosurgery clinic. Researchers had access to all medical records, including the results of specialist consultations, but the most important data were our own based on the performed tests and scales.

### 2.3. Statistical Analysis

Normal distributions of the study variables were verified with the Shapiro–Wilk test. All data are presented as mean ± SD or number (percentage) participants. Multiple groups were compared by analysis of variance (ANOVA) followed by Tukey’s HSD test or by the Kruskal–Wallis rank-sum test. Differences in quantitative variables were determined with the use of a parametric t-test or a nonparametric Mann–Whitney test. Relationships between categorical variables were determined with Pearson’s chi-squared test. To investigate group and time effects in functional activity, we used a multiple repeated measures analysis of variance (ANOVA) between groups (CH group, VS group, CPA group, and control group) and with time (before surgery/after surgery/at discharge). A *p*-value < 0.05 was considered statistically significant. All calculations were carried out using the Statistica 13.0 PL statistical package (StatSoft, Kraków, Poland).

## 3. Results

Of 835 patients with brain tumors, the majority (649, 77.7%) had CH surgery, 96 of whom (14.8%) required rehabilitation; 53 patients (6.3%) had VS tumors, 16 (30.2%) of whom needed postoperative rehabilitation; and 52 (6.2%) patients had CPA tumors, 15 (28.8%) of whom required rehabilitated. There were 81 patients (9.8%) who had tumors at other locations that were not included due to the small sample sizes at each tumor location.

Eighty patients after CH tumor surgery (12.3%) required rehabilitation due to limb paresis or paralysis compared with 26.4% patients after VS tumor surgery, and only one patient after CPA tumor surgery. Thirteen patients (25.0%) after CPA tumor surgery, five patients (0.8%) with CH tumors, and one patient with a VS tumor (1.8%) received rehabilitation for facial nerve palsy. Other reasons for rehabilitation included balance and coordination disturbances, general weakness disallowing independent functioning, and apraxia (20 people in total).

Six hundred and eighty-five patients (82%) had received first surgery: 518 for CH tumors, 49 for VS tumors, 46 for CPA tumors, and 72 for tumors at other locations. One hundred and fifty patients had received reoperations (18%): 131 for CH tumors, 4 for VS tumors, 6 for CPA tumors, and 9 at other locations. The proportion of first operation and reoperations was similar for all patients (Table 1) and rehabilitated patients (Table 2). The VS group was characterized by significantly longer rehabilitation, overall LOS, and postoperative LOS compared with the CH and CPA groups (all *p* < 0.001). Similarly, the VS group spent longer in ICU after surgery compared with the CH group (Table 2).

Complications affected the course of rehabilitation in 35 patients. Twenty-eight patients had a single complication, four had two complications, and three had three complications. The most common complications were bleeding into the VS and hydrocephalus (seven patients); hematoma, cerebrospinal fluid leakage from the surgical wound, and cardiorespiratory failure in six people; and edema in four people. These complications occurred in 21/96 (21.9%) patients in the CH group (3.2% of all 649 operated CH tumors); 13/16 (81.3%) patients in the VS group (24.5% of all 53 operated VS tumors); and 1/15 (6.7%) patients in the CPA group (1.9% of all 52 operated CPA tumors). Patients in the VS group had a particularly high complication rate, especially Grade II and III complications, and a higher proportion of surgical complications than medical complications (Table 3).

Two-way repeated ANOVA revealed a difference between groups at different timepoints (before surgery, after surgery, and at discharge) for BI, KPS, MRS, and GI (all *p* < 0.001). There was a significant interaction between group and timepoint, indicating that the effects of time were different for the four groups (Table 4). After surgery, VS, CH, and CPA groups showed significantly lower BI, KPS, and GI scores and higher MRS scores compared with before surgery and at discharge. At discharge, CH, VS, and CPA groups were characterized by higher BI, KPS, and GI values and lower MRS values compared with after surgery (*p* < 0.001). The control group had comparable scores for each instrument, and no significant differences were observed (all *p* > 0.05).

The mean BI score in the CH and CPA groups classified patients as independent before surgery (BI > 80), while the patients in the VS group required help (BI = 77.5). After surgery, patients in the CH and CPA groups required help to various degrees (BI, 21–80), with VS group patients classified as “severe” (BI = 20). Patients in the CPA group returned to pre-surgery levels (independent) at discharge, while patients in the CH and VS groups required help to varying degrees. The control patients in all three assessments were independent according to the BI scale (Table 4).

The mean KPS score before surgery in the CH, VS, and CPA groups classified patients as able to carry on normal activity and work, with no special care needed (KPS > 70). The mean KPS decreased after surgery and the CH and CPA patients were classified as unable to work, able to live at home, care for most personal needs, with a varying degree of assistance needed (KPS 40–70), while the VS patients were unable to care for self (KPS = 35). KPS values at discharge for the CH and VS groups were between 40 and 70, and CPA patients returned to a score > 70. The control patients in all three assessments were classified as able to carry on with normal activities (KPS > 70) (Table 4).

The mean MRS score in the CH and VS groups classified patients as slightly disabled: unable to perform all previous activities but able to look after their own affairs without assistance. Patients in the CPA group were not significantly disabled (MRS = 0.7). After surgery, patients in the VS group had the highest score (MRS = 4.5), denoting severe disability: bedridden, incontinent, and requiring constant nursing care and attention. The scores in CH and CPA patients (3.4, 2.9) meant moderate disability. Patients in the CPA group returned to pre-surgery levels (no significant disability) at discharge, while patients in the CH and VS groups were classified as moderately disabled. In all three assessments, the control patients were not significantly disabled and, despite symptoms, were able to carry out all usual duties and activities (Table 4).

The mean GI score in the CH and VS groups classified patients as independently walking with orthopedic equipment before surgery (GI 7–8), while patients in the CPA group walked independently (GI = 9.5). After surgery, patients in the CH and VS groups could not walk (GI < 5), while CPA patients could walk with the assistance of another person but only within a room and with access to a wheelchair (GI = 5.2). Patients in the CPA group returned to pre-surgery levels (independent gait) at discharge, patients in the CH group could walk with orthopedic equipment, and VS group patients walked with the assistance of another person, but only within a room. The control patients were independent in gait in all three assessments (Table 4).

**Figure 1:**
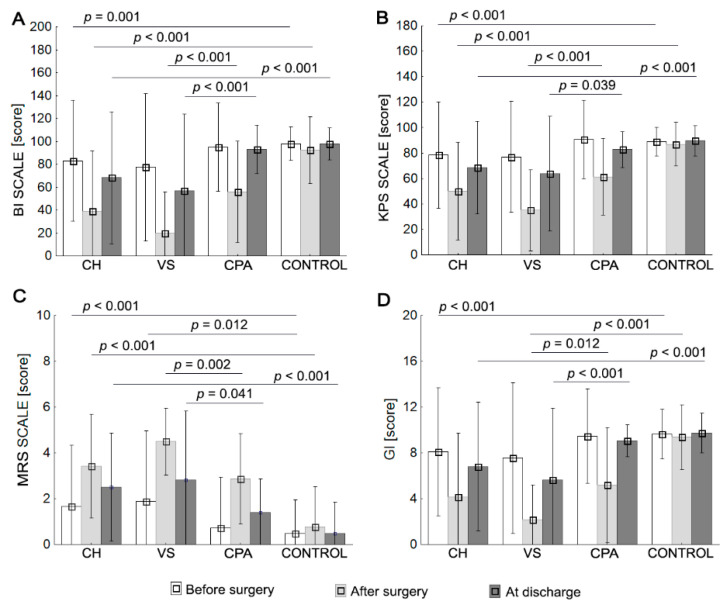
Patient groups’ (CH, cerebral hemisphere group; VS, ventricular system group; CPA, cerebellopontine angle group; control group) mean values (±SD) before surgery, after surgery, and at discharge for BI (**A**); KPS (**B**); MRS (**C**); and GI (**D**).

The CH and VS groups had significantly lower BI, KPS, and GI scores and higher MRS scores than the control group before surgery. After surgery and at discharge, the CPA group was characterized by higher BI, KPS, and GI scores and lower MRS scores compared to the CH group, respectively (*p* < 0.05; Table 4 and Figure 1).

The proportion of patients walking independently (GI > 6) decreased by 24% in the CH group and by 31.3% in the VS group on the day of discharge compared with before surgery. The percentage of self-walking patients increased by 6.7% in the CPA group and by 2.5% in the control group (Table 5). Table 5 shows the functional state before surgery, after surgery, and at discharge (*n* = 205).

**Figure 2:**
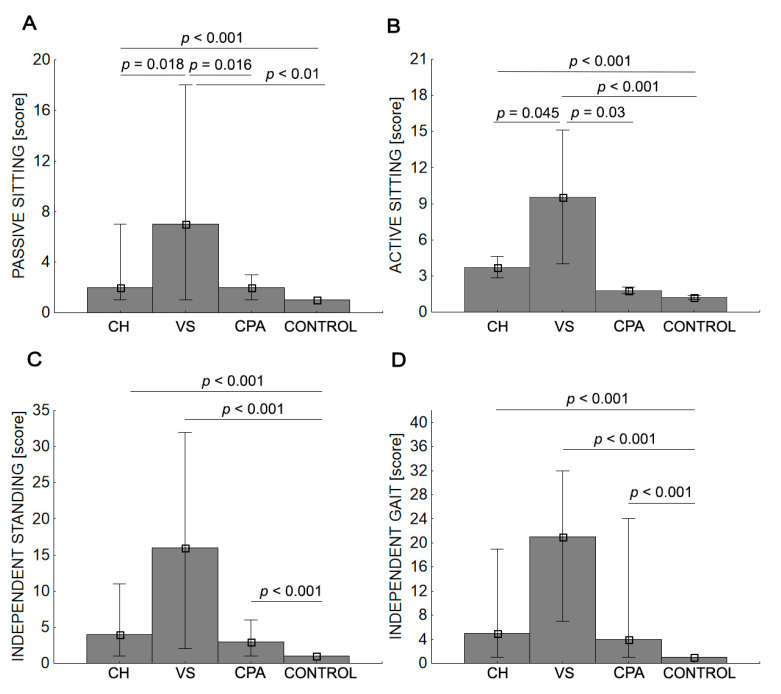
Average time (in days) to obtaining the evaluated functional capabilities: passive sitting (**A**); active sitting (**B**); independent standing (**C**); independent gait (**D**). CH, cerebral hemisphere group; VS, ventricular system group; CPA, cerebellopontine angle group.

The VS group was characterized by significantly longer time taken to passively sit and actively sit compared with the CPA, CH, and control groups after surgery (<0.05). VS, CPA, and CH groups were comparable with respect to independent standing and gait (all *p* > 0.05). The control group was characterized by significantly shorter times for all conditions (Table 6, Figure 2). The mean values given in Table 6 were calculated not for whole groups but only for those patients who had achieved particular motor skills at discharge.

## 4. Discussion

Organization of the care and rehabilitation of patients after brain tumor surgery depends on many factors. One of the most important determinants of the course of postoperative treatment is the tumor location and the related surgical approach. Our primary aim was to assess functional status, motor skills, and gait efficiency in patients undergoing surgery for brain tumors. An additional aim was to assess the incidence of complications affecting the rehabilitation course and time parameters including overall LOS, LOS after surgery, LOS in the ICU, the time needed for rehabilitation, and the time needed to improve the loss of basic motor skills after surgery. Our results showed that patients with VS tumors, compared with those with CH and CPA tumors, had the worst motor function and independence in daily activities (before surgery, immediately after operation, and at discharge) according to all metrics. Patients with VS tumors had a higher proportion of surgical complications, most often required postoperative rehabilitation, and their rehabilitation lasted the longest, resulting in the longest hospital stay.

Despite continuous progress in diagnosis and treatment [32,33], patients with brain tumors still have a high occurrence of postoperative motor deficits (9–42%, depending on the method of evaluation) [2,34,35,36,37]. Here, 16.6% of 835 patients operated on for brain tumors required rehabilitation due to neurological complications, and 14.8% of patients with CH tumors required rehabilitation, including 12.3% for limb paresis or paralysis (4.7% experiencing new motor deficits). Balance and coordination disturbances, apraxia, and other complications affecting motor function were present in 2.3% of cases. Fifty-three patients (6.3%) were treated for VS tumors, 30.2% of whom needed postoperative rehabilitation: 26.4% for motor deficits (15.1% new motor deficits) and 7.3% for weakness, ataxia, and balance disorders. Aftahy et al. [15] similarly reported 26.6% new neurological deficits after VS surgery. Although the proportion of patients requiring rehabilitation after surgery for CPA tumors was as high as for patients with VS tumors, the reasons for rehabilitation were completely different. Only one patient needed physical therapy for limb paresis, but one in four patients had facial nerve palsy (5.8% before surgery). The prevalence of cranial nerve VII paresis varies and is reported in 20–70% of patients after vestibular schwannoma removal and 10–40% patients after surgery for CPA meningiomas [11,13,38]. However, surgical techniques usually allow for total resection of these tumors, which are usually benign and rarely transform into malignant variants [39].

Perioperative complications also impact the results and course of rehabilitation and are reported in anywhere between nine and 40% of patients [40], with a mode of around 20% [41,42,43,44,45]. Postoperative hemorrhage, hematoma, and edema often require reoperation and involve a prolonged ICU stay [40,41,46,47]. Hydrocephalus, bleeding into the ventricular system, and CSF leak involve staying recumbent for a few to several days for recovery (due to ventricular or lumbar drain and ventriculoperitoneal shunt) [41]. In our study, complications were rare in the CPA group, and the most common and most severe complications were in the VS group, predominantly Grade II and III according to the Landriel Ibañez classification. In the CH group, the most common complications were Grade I and II, and complications directly related to surgery were more common than medical complications in all groups. Patients who developed a surgical complication had significantly longer LOS, total hospital costs, and higher rates of other complications. Complications are a negative prognostic factor and delay starting follow-up treatment. Furthermore, new neurological deficits are associated with decreased overall survival [34,36,40,46,48].

Previous studies analyzing the impact of a complication on LOS have usually either focused on a specific tumor type or specific surgical method [42,49,50]. Others have assessed the impact of general brain tumors or craniotomy on hospital stay [51,52,53]. Our analysis provides new insights into the impact of tumor location on LOS. VS tumors resulted in the longest LOS of all tumors; both the overall LOS (39.2 days) and LOS after surgery (34.6 days) were on average about 20 days longer than for patients with CH and CPA tumors, which were similar. This may be surprising given that the pre- and postoperative condition, percentage of patients with limb paresis, and the proportion of patients with severe complications were significantly worse in the CH group. The LOS of patients with CPA tumors was prolonged mainly due to facial nerve palsy. In the control group, the LOS after surgery was 5.1 days. While our data are consistent with data from other centers [49,50,51,52,53], there is an increasing trend, especially in the US, to reduce the LOS to one day by using the awake-surgery method, which increases safety without compromising outcomes [42,49]. Time in hospital or spent rehabilitating becomes particularly important in patients with malignant brain tumors, since their progression-free and overall survival are usually of the order of only a few months. Another metric affected by inpatient stay in cancer patients is Overall Survival Outside Hospital [50].Tumor treating fields (TTFs) is a newer therapy that seems to effectively improve this parameter and has been used since 2011 as salvage therapy for recurrent glioblastoma and, since 2015, for newly diagnosed glioblastoma. The current evidence supports the use of TTFs as an efficacious, antimitotic treatment with minimal toxicity in patients with glioblastoma [33,54]. We examined four levels of motor activity. The lowest level was passive sitting, which means that patients can spend time in a wheelchair. The second level was active sitting, which has been shown to have prognostic value as demonstrated by its strong correlation with ADL; patients who can sit without support soon perform better in functions of everyday life [23,55]. The average time needed to obtain trunk control in a sitting position was approximately three times longer in the VS group than the CH group and five times longer than in the CPA group. This position was still unattainable at discharge in 9.4% of all patients with VS tumors, but all CPA and control patients and 99.1% of CH patients achieved a sitting position. The third level of performance assessed, independent standing, greatly increases participation in ADL [23,56], and the fourth level of motor skill, independent gait, not only determines participation in an active life and provides a sense of self-confidence but is also important for social and mental health. Gait efficiency, especially speed of gait, is a known survival factor in patients with brain tumors [57]. We found that gait efficiency was worst in the VS group, both before and after surgery. The proportion of VS tumor patients who could not walk increased from 9.4% at admission to 18.9% at discharge, compared with 2.6% to 6.2% for CH tumors and 1.9% to 0 for CPA tumors. The average time it took to achieve independent gait was more than twice as long in the VS group than in the other two groups.

Each scale used here assessed a different aspect of activity: KPS, the performance status in the context of cancer; MRS, the degree of dependence on other people; and BI, independence in ADL. These scales have been used by some authors to predict postoperative function and hospital discharge time [49,58,59]. Others have reported the association between functional status assessed on the BI [60] and MRS [61] scales and overall survival. Our study differs from previous studies in that we added our own GI scale to present a comprehensive overview of the condition of our patients, since gait re-education is often a main goal of postoperative rehabilitation. The second difference is that we assessed functional status immediately after surgery in the acute period. While intuitively patients may feel a deterioration in well-being due to the operation, the tumor location—and therefore the method of surgery, duration, access, and other factors—also affects patients in the early postoperative period. Cinotti et al. [62] showed that the preoperative functional state is a predictor of postoperative neurologic complications. We found a similar relationship. The lowest values for all four indicators before surgery and the largest decrease in the values in the early postoperative period were found in patients with VS tumors. The course of rehabilitation was disturbed by more frequent neurological deficits and complications, which resulted in the worst metrics at discharge in this group. The CH group had better results for all four scales and at all three evaluation timepoints compared to the VS group but worse than the CPA group. The CPA group was similar to the control group at admission and discharge but significantly worse in the early postoperative period. In the control group, all the scores decreased only slightly, and even in the first few days after surgery, the difference was not significant. These control data support the approach of US neurosurgeons seeking to shorten the LOS of some operated patients to one day [42,49].

Our results are unequivocal due to several factors. First, VS tumors can disrupt the functions of brain regions important to the whole organism (the fourth ventricle floor, the thalamus). Second, an intraventricular location is always a deep site. When a tumor cannot be removed endoscopically, access to it often poses a risk of greater damage to the brain tissue located in the access path compared to other locations. Different surgical approaches to the ventricles can be used, namelythe telovelar approach, the transvermian trajectory, and the tonsillobiventral fissure approach. The selection of the surgical approach for intraventricular tumor resection fundamentally depends on the surgeon’s experience and preference. Third, hemostasis during VS tumor surgery is difficult as some agents cannot be used in the ventricular system and due to reduced blood clotting in the CSF environment. Fourth, patients receiving surgery at this site frequently need a postoperative ventricular drain, which increases the risk of CNS infection and prolongs the ban on verticalization and delays the start of intensive rehabilitation. Finally, these tumors often result in hydrocephalus, which may require further surgical intervention [4,15,16,17,63,64].

Our study has some limitations. First, the group sizes were very different (reflecting the incidence of tumors at these locations); thus, despite the clear differences between groups, there may be bias. Our study was single-center and, although covering 1.5 years and involving 835 patients, represents a relatively small number of patients, since only 16.6% required postoperative rehabilitation. The tumor size, histology, type, and precise location were unknown, and these parameters would be interesting to study with respect to function. The surgical approach was another important factor to consider, but we did not analyze this on the assumption that in each individual case, the choice was optimal. Finally, we did not use information on some important neurological parameters such as the degree of paresis before and after surgery, which was beyond the scope of this article. Future studies should also assess the impact of complications arising from first surgery or re-operation, a subgroup we excluded due to a lack of statistical power.

## 5. Conclusions

Patients receiving operations for VS tumors had the worst motor function and independence in daily activities before surgery, and this group deteriorated both immediately after operation and at discharge according to all metrics. Postoperative complications were also most common in this group. Patients with VS tumors most often required postoperative rehabilitation, and their rehabilitation lasted the longest, which resulted in the longest hospital stay. VS tumor patients represent a rehabilitation challenge in a neurosurgical unit, and postoperative rehabilitation planning must take the tumor site and preoperative condition into account.

## Figures and Tables

**Table 1:** Reasons for rehabilitation in patients undergoing surgery for tumors of the cerebral hemisphere (CH), ventricular system (VS), and cerebellopontine angle (CPA) (*n* = 754).

	CH		VS		CPA	
	*n* = 649		*n* = 53		*n* = 52	
*n* (%)	Prior to surgery	After surgery	Prior to surgery	After surgery	Prior to surgery	After surgery
Limb paresis or paralysis	49 (7.6%)	80 (12.3%)	6 (11.3%)	14 (26.4%)	1 (1.9%)	1 (1.9%)
Facial nerve palsy	2 (0.3%)	5 (0.8%)	0	1 (1.8%)	3 (5.8%)	13 (25.0%)
Balance disturbances, weakness, other	-	15 (2.3%)	-	4 (7.3%)	-	1 (1.9%)
Postoperative complications	-	21 (3.2%)	-	13 (24.5%)	-	1 (1.9%)
First surgery	518 (79.8%)		49 (92.5%)		46 (88.7%)	
Re-operation	131 (20.2%)		4 (7.5%)		6 (11.3%)	

Abbreviations: CH, cerebral hemisphere group; VS, ventricular system group; CPA cerebellopontine angle group.

**Table 2:** Demographic characteristics of the study participants and time parameters of treatment (*n* = 205).

	CH *n* = 96	VS *n* = 16	CPA *n* = 15	Control *n* = 78	*p*-Value
Male *n* (%) Female *n* (%)	51 (53.1%) 45 (46.9%)	8 (50%) 8 (50%)	6 (40%) 9 (60%)	31 (39.7%) 47 (60.3%)	0.328
Age mean ± SD, [range]	53.0 ± 15.3 [19–83]	38.9 ± 15.5 [21–81]	41.7 ± 15.2 [23–71]	46.8 ± 14.6 [22–78]	0.003
First surgery *n* (%)	70 (72.9%)	15 (93.8%)	13 (86.7%)	64 (82.0%)	0.157
Re-operation *n* (%)	26 (27.1%)	1 (6.2%)	2 (13.3%)	14 (18.0%)	
Overall LOS (days)	18.3 ± 10.7 [4–52]	39.2 ± 22.1 [12–92]	15.1 ± 6.9 [8–32]	8.6 ± 2.0 [5–14]	<0.001
LOS after surgery (days)	14.7 ± 10.3 [2–50]	34.6 ± 22.3 [10–90]	12.1 ± 6.8 [5–28]	5.1 ± 1.1 [3–8]	<0.001
Days in ICU after surgery	0.7 ± 3.6 [0–31]	3.9 ± 10.0 [0–40]	-	-	0.022
Days of rehabilitation	11.2 ± 8.2 [1–42]	25.2 ± 14.2 [8–58]	8.9 ± 6.0 [3–23]	-	<0.001

Abbreviations: CH, cerebral hemisphere group; VS, ventricular system group; CPA cerebellopontine angle group; LOS, the length of stay; ICU; intensive care unit.

**Table 3:** Complications affecting the course of rehabilitation (the Landriel Ibañez Classification) (*n* = 127).

	CH *n* = 96	VS *n* = 16	CPA *n* = 15	*p*-Value
Patients with complications *n* (%)	21 (21.9%)	13 (81.3%)	1 (6.7%)	0.001
Number of complications (*n*)	27 *	17 *	1 *	
Grade I *n* (%)	10 (10.4%)	3 (18.8%))	0	0.226
Grade II *n* (%)	10 (10.4%)	9 (56.3%)	1	<0.001
Grade III *n* (%)	7 (7.3%)	5 (31.3%)	0	0.004
Surgical/medical *n* (%)	19/8 (70%/30%)	14/3 (82%/18%)	1/0	0.565
Temporary/permanent *n* (%)	16/11 (59%/41%)	11/6 (65%/35%)	0/1	0.435

Abbreviations: CH, cerebral hemisphere group; VS, ventricular system group; CPA cerebellopontine angle group. * Twenty-eight patients had a single complication, four patients had two complications, and three patients had three complications.

**Table 4:** Activities of daily living, performance, self-reliance, and gait efficiency before surgery, after surgery, and at discharge (*n* = 205).

Variable	Time	CH *n* = 96	VS *n* =16	CPA *n* = 15	Control *n* = 78
		Mean ± SD [Range]	Mean ± SD [Range]	Mean ± SD [Range]	Mean ± SD [Range]
BI	Before surgery	83.2 ± 26.4 [5–100]	77.5 ± 32.2 [15–100]	95.0 ± 19.4 [25–100]	98.1 ± 7.3 [55–100]
	After surgery	38.9 ± 26.3 [0–100]	20.0 ± 17.9 [0–60]	56.0 ± 22.2 [25–100]	92.4 ± 14.5 [45–100]
	At discharge	68.3 ± 28.8 [0–100]	56.9 ± 33.5 [0–100]	93.0 ± 10.7 [65–100]	98.0 ± 7.0 [55–100]
KPS	Before surgery	78.4 ± 10.9 [20–100]	76.9 ± 21.8 [30–100]	90.7 ± 15.3 [40–100]	89.1 ± 5.6 [60–100]
	After surgery	50.1 ± 19.2 [10–90]	35.0 ± 15.9 [10–60]	61.3 ± 15.1 [40–90]	86.9 ± 8.6 [60–100]
	At discharge	68.6 ± 18.3 [10–100]	63.8 ± 22.5 [20–90]	82.7 ± 7.0 [70–90]	89.6 ± 5.9 [60–100]
MRS	Before surgery	1.7 ± 1.3 [0–5]	1.9 ± 1.5 [0–5]	0.7 ± 1.1 [0–4]	0.5 ± 0.7 [0–3]
	After surgery	3.4 ± 1.1 [0–5]	4.5 ± 0.7 [3–5]	2.9 ± 1.0 [1–4]	0.8 ± 0.9 [0–3]
	At discharge	2.5 ± 1.2 [0–5]	2.8 ± 1.5 [1–5]	1.4 ± 0.7 [0–3]	0.5 ± 0.7 [0–3]
GI	Before surgery	8.1 ± 2.8 [1–10]	7.6 ± 3.3 [1–10]	9.5 ± 2.1 [2–10]	9.6 ± 1.1 [4–10]
	After surgery	4.2 ± 2.8 [1–10]	2.2 ± 1.5 [1–5]	5.2 ± 2.5 [1–10]	9.4 ± 1.4 [3–10]
	At discharge	6.8 ± 2.8 [1–10]	5.6 ± 3.1 [1–10]	9.1 ± 0.7 [7–10]	9.7 ± 0.9 [4–10]

*p*-value: Difference between groups at different timepoints (before surgery, after surgery, and at discharge) for BI, KPS, MRS, and GI in two-way repeated ANOVA, all *p* < 0.001. Abbreviations: CH, cerebral hemisphere group; VS, ventricular system group; CPA cerebellopontine angle group; BI, Barthel Index; KPS, Karnofsky Performance Scale; MRS, modified Rankin scale; GI, Gait Index; SD, standard deviation.

**Table 5:** Functional state before surgery, after surgery, and at discharge (*n* = 205).

Motor Skills	CH *n* = 96	VS *n* = 16	CPA *n* = 15	C *n* = 78
	*n* (%)	*n* (%)	*n* (%)	*n* (%)
Before surgery				
Passive sitting	95 (99.0%)	16 (100%)	15 (100%)	78 (100%)
Active sitting	93 (96.9%)	16 (100%)	15 (100%)	78 (100%)
Standing	84 (87.5%)	12 (75.0%)	14 (93.3%)	77 (98.7%)
Independent gait	79 (82.3%)	11 (68.8%)	14 (93.3%)	74 (94.9%)
Week after surgery				
Passive sitting	89 (92.7%)	11 (68.8%)	15 (100%)	78 (100%)
Active sitting	80 (83.3%)	5 (31.3%)	15 (100%)	78 (100%)
Standing	62 (64.6%)	2 (12.5%)	13 (86.7%)	78 (100%)
Independent gait	37 (38.5%)	1 (6.3%)	11 (73.3%)	76 (97.4%)
At discharge				
Passive sitting	96 (100%)	15 (93.6%)	15 (100%)	78 (100%)
Active sitting	90 (93.8%)	11 (68.8%)	15 (100%)	78 (100%)
Standing	80 (83.3%)	10 (62.5%)	15 (100%)	78 (100%)
Independent gait	56 (58.3%)	6 (37.5%)	15 (100%)	76 (97.4%)

Abbreviations: CH, cerebral hemisphere group; VS, ventricular system group; CPA, cerebellopontine angle group; C, control group.

**Table 6:** Average time (days) to obtaining the evaluated functional capabilities (*n* = 205).

After Surgery	CH	VS	CPA	Control
(Days)	Mean ± SD [Range]	Mean ± SD [Range]	Mean ± SD [Range]	Mean ± SD [Range]
Passive sitting	3.8 ± 7.2 [1–66]	7.4 ± 5.8 [7–15]	1.7 ± 0.6 [1–3]	1.2 ± 0.5 [1–4]
Active sitting	3.7 ± 4.2 [1–23]	9.5 ± 9.2 [2–32]	1.8 ± 0.6 [1–3]	1.2 ± 0.6 [1–4]
Independent standing	5.9 ± 6.7 [1–45]	17.1 ± 10.2 [2–32]	4.9 ± 5.0 [1–21]	1.3 ± 0.7 [1–4]
Independent gait	7.0 ± 6.7 [1–26]	20.0 ± 9.3 [7–32]	7.3 ± 6.8 [1–24]	1.5 ± 1.0 [1–4]

*p*-value: Post hoc tests for differences between groups for average time needed to obtain the possibility of passive sitting, active sitting, independent standing, and gait are presented in Figure 2.

## Data Availability

All the data are presented within the manuscript.
